# Omphalocele and Gastroschisis Associated With Multiple Congenital Abnormalities

**Published:** 2011-08-25

**Authors:** R Singal, LN Garg, RP Singal, S Gupta, SR Shahi, S Singal, B Singh

**Affiliations:** * Department of Surgery, Maharishi Markandeshwer Institute of Medical Sciences and Research, Mullana, Distt–Ambala, HaryanaIndia; ** Department of Otolaryngology (ENT), Maharishi Markandeshwer Institute of Medical Sciences and Research, Mullana, Distt–Ambala, Haryana India; *** Department of Orthopedics, C/o Dr. Kundan Lal Hospital, Ahmedgarh, District: Sangrur (Punjab) India; **** Department of Radiology, Maharishi Markandeshwer Institute of Medical Sciences and Research, Mullana, Distt–Ambala, Haryana India; ***** Dept of Medicine and Ayurvedic, C/o Dr Kundan Lal Hospital, Multispecialty Hospital, Ahmedgarh, Distt–Sangrur, Punjab India; ****** Dept of Gynecology, Multispecialty Hospital, C/o Dr Kundan Lal Hospital, Ahmedgarh, Distt– Sangrur, Punjab India; ******* Department of Surgery, Maharishi Markandeshwer Institute of Medical Sciences and Research, Mullana, Distt–Ambala, Haryana India

**Keywords:** multiple abnormalities, cyanosis, imperforate anus, musculoskeletal deformity

We are reporting a rare case of a newborn baby diagnosed with an abdominal wall defect together with multiple congenital abnormalities. Mother delivered a dead male baby, diagnosed as gastroschisis. There were multiple defects seen as spinal deformity, imperforate anus, esophageal fistula and lower limb deformity (congenital talipes equinovarus) along with the webbing of neck (Figure 1 and Figure 2). There were also ischemic changes present over the left upper limb in the form of cyanosis. The diagnosis made was gastrochisis and Omphalocele along with spinal deformity.

**Figure 1:**
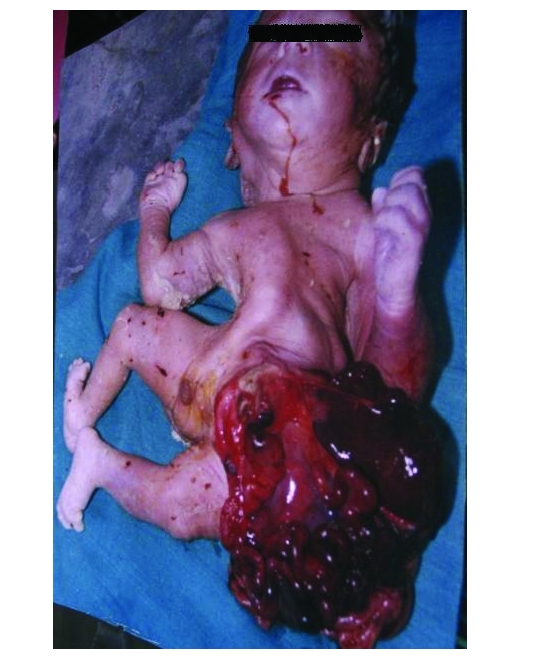
Showing multiple defects and gastrochisis with cyanosis of the left hand

**Figure 2:**
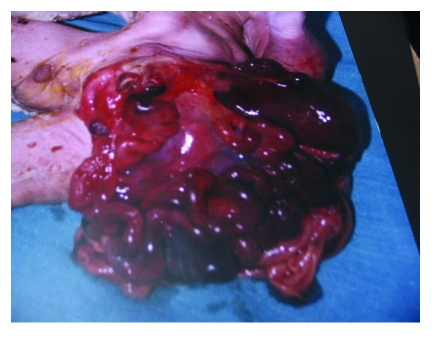
Showing multiple defects and gastrochisis with cyanosis of the left hand

Gastroschisis is a congenital full–thickness defect of the anterior abdominal wall, whose incidence is increasing [1]. Omphalocele is a congenital defect of the abdominal wall and it is estimated to occur in approximately 1 in 6000 births [2, 3]. The cause of gastrochisis is presently unknown, but a prevailing theory is that it results from an abdominal wall defect associated with normal involution of the second umbilical vein [1].

The term VATER or VACTERL syndrome used as (V– vertebral, A – anal, C–cardiac, TE– tracheal–esophageal fistula, R– renal, L–limb). Scoliosis (sko–lee–oh–sis) is a term coming from a Greek word meaning curvature. The disease causes the spine to curve laterally (to the side) usually in the shape of an ‘S’ or ‘C’ as seen in our case. Kyphosis (ky–foe–sis) is the normal curvature of the rib–bearing thoracic spine. Excessive kyphosis may develop as a result of a poor posture early in life. Kyphosis means the spine is bent forward.

In this context, Duhamel introduced the syndrome of caudal regression, defined as a continuum of anorectal, urogenital and skeletal congenital abnormalities, with the sirnomelia and imperforate anus. The incidence of anorectal malformations varies between 1 in 4000 and 1 in 5000 live births. The male predominance is of 1.5%. In congenital idiopathic clubfoot (i.e., talipes equino–varus), the infant's foot points downward (i.e., equinus) and turns inward (i.e., varus), while the forefoot curls toward the heel (i.e., adduction) [4]. 

In children with gastrochisis, life threatening associated anomalies occurs less often than in children with omphalocele, but conditions associated with prematurity, intestinal atresis, and short bowel syndrome may add significantly to their morbidity. 

There were multiple congenital abnormalities associated with musculoskeletal deformity in our case, cyanosis of the left hand, webbing of the neck, which is very rare. Most spinal defects occur at the level of the lumbar vertebrae with the sacral, thoracic and cervical areas, in decreasing order of frequency. Cyanosis, associated with an omphalocele, implies either an intracardiac malformation, or, perhaps, an associated diaphragmatic hernia, as in our case, as cyanosis was present. Spinal bony abnormalities range in incidence form 30% to 44% varies according to anal lesions. Scoliosis is associated with arthrogryposis (2.5% to 34% incidence) [5]. The true etiology of congenital clubfoot is unknown. Cytogenic abnormalities (e.g. – congenital talipes equinovarus (CTEV) deformity can be seen in syndromes involving chromosomal deletion. Incidence of this in the general population is 1 per 1000 live births. If the child is alive, then the child should undergo a proper evaluation, as the latest investigation is MRI with X–rays or pre–natal checkup.

## References

[1] Dabbas N, Muktar Z (2009). GABBY: An ex vivo model for learning and refining the technique of preformed silo application in the management of gastroschisis. Afr J Paediatr Surg.

[2] Kazaura MR (2004). Increasing Risk of Gastrochisis in Norway: An Age–Period–Cohort Analysis. Am J Epidemiol.

[3] Balc S, Leblebicioğlu G (2005). A new case of omphalocele with absence of thumb. Turk J Pediatr.

[4] Tanna GLD, Rosano A, Mostroicovo P (2002). Prevalence of gastroschisis at birth: retrospective study. BMJ.

[5] Loder RT, Guiboux JP (1993). Musculoskeletal Involvement in Children with Gastroschisis and Omphalocele. Journal of Pediatric Surgery.

